# Patients with Crohn’s Disease Achieving Ustekinumab-Induced Remission Are Characterized by Increased Baseline IL-23 Receptor Expression on Lamina Propria Th1 Cells

**DOI:** 10.3390/jcm15145434

**Published:** 2026-07-10

**Authors:** Sara Onali, Amalia di Petrillo, Agnese Favale, Rita Pillai, Massimo Claudio Fantini

**Affiliations:** 1 Department of Medical Science and Public Health, University of Cagliari, 09124 Cagliari, Italy; sara.onali@unica.it (S.O.); amalia.dip@unica.it (A.d.P.); agnese.favale@unica.it (A.F.); 2 Center for Research University Services-CeSAR, University of Cagliari, 09124 Cagliari, Italy; rpillai@unica.it

**Keywords:** Crohn’s disease, ustekinumab, mucosal immunity

## Abstract

**Background/Objectives:** Ustekinumab, targeting the shared p40 subunit of interleukin (IL)-12 and IL-23, is an effective therapy for Crohn’s disease (CD), yet reliable predictors of response remain lacking. Given the central role of the IL-12/IL-23 axis in intestinal inflammation, we aimed to characterize the baseline mucosal expression of IL-12/IL-23 pathway components in lamina propria immune cells, and to explore their association with clinical response and remission following ustekinumab therapy. **Methods:** In this prospective, single-center study, biopsy-derived lamina propria mononuclear cells (LPMCs) were obtained from patients with CD prior to ustekinumab initiation. Gene expression of IL-12/IL-23 cytokine subunits and receptors was assessed by quantitative real-time PCR. Flow cytometry was performed to evaluate the distribution of T helper and innate lymphoid cell subsets and the expression of IL-23R and IL-12Rβ2. Clinical outcomes were assessed at week 16. **Results:** Fifteen consecutive patients were enrolled and included in the study. At week 16, 14/15 (93.3%) and 9/15 (60.0%) of patients reached clinical response and remission, respectively. No statistically significant differences in baseline mucosal gene expression of IL-12/IL-23 pathway components were observed between remitters and non-remitters. A trend toward higher expression of receptor subunits (IL23R, IL12RB1, IL12RB2) was observed in remitters, albeit with high variability and overlapping distributions. Similarly, cytokine subunits (IL23p19, IL12/IL23p40, IL12p35) showed no consistent differential expression pattern between the groups. In contrast, flow cytometry revealed a significantly higher frequency of IL-23R-expressing Th1 cells in remitters compared with non-remitters (20.6% vs. 6.8%, *p* = 0.009). **Conclusions:** Baseline transcriptional profiling of IL-12/IL-23 pathway components was not associated with remission following ustekinumab therapy. However, increased expression of IL-23R on mucosal Th1 cells identified a distinct immunological signature associated with clinical remission, suggesting that IL-23R expression on mucosal Th1 cells may represent a promising candidate biomarker that requires validation in larger independent cohorts.

## 1. Introduction

Crohn’s disease (CD) is a chronic, relapsing inflammatory disorder of the gastrointestinal tract characterized by a dysregulated immune response against luminal antigens. The pathogenesis of CD is sustained by an aberrant activation of innate and adaptive immune pathways, with a central role for T helper (Th) cell-mediated inflammation, particularly Th1 and Th17 responses. The IL-12/IL-23 axis represents a pivotal immunological pathway driving intestinal inflammation through the expansion and stabilization of pathogenic effector T cells [1].

Tumor necrosis factor-α (TNF-α) antagonists have long represented the cornerstone of biological therapy in moderate-to-severe CD. However, up to 30–40% of patients fail to respond to induction therapy, and a substantial proportion experience secondary loss of response over time [2]. Growing evidence suggests that failure of anti-TNF therapy may reflect a shift toward alternative inflammatory pathways, particularly those mediated by IL-23 [3]. In this context, IL-23 has emerged as a key cytokine in sustaining chronic intestinal inflammation and promoting the pathogenicity of effector T cell subsets.

Ustekinumab, a fully human monoclonal antibody targeting the p40 subunit shared by IL-12 and IL-23, has demonstrated efficacy in both anti-TNF-naïve and anti-TNF-experienced patients with CD. While the clinical effectiveness of ustekinumab is well established, the immunological mechanisms underlying differential patient responses remain incompletely understood [4]. In particular, little is known about the mucosal immune profiles associated with clinical remission following IL-12/IL-23 blockade.

Several studies have attempted to identify predictors of response to ustekinumab by analyzing serologic biomarkers. Recently, exploratory studies have investigated serum cytokine profiles and pharmacokinetic parameters as potential predictors of therapeutic outcome. In a prospective pilot study, baseline serum IL-23 levels were found to correlate with mucosal healing following ustekinumab therapy, suggesting a possible role for circulating IL-23 as a biomarker of response [5]. In addition, longitudinal analyses of serum cytokines during induction have shown distinct dynamic patterns between responders and non-responders, including differential modulation of IL-13, IL-8 and IL-6, as well as higher ustekinumab trough levels in responders [6].

However, these studies were limited by measures exclusively performed in peripheral blood measurements, without direct assessment of the intestinal mucosal immune compartment. Given that intestinal inflammation is driven by tissue-specific immune responses driven by locally infiltrating cells and that serological biomarkers may not accurately reflect mucosal immunopathology, the clinical utility of serum-based predictors remains uncertain. Moreover, they did not investigate the composition and functional heterogeneity of IL12/IL23-responding mucosal immune cell subsets, which are known to play a central role in CD pathogenesis. To date, no validated biomarker is available to reliably predict response or remission following ustekinumab therapy, and the mucosal immune signatures underlying differential therapeutic outcomes remain largely unexplored [7].

Therefore, the aim of this study was to analyze the expression of IL-12 and IL-23 and their specific receptors on lamina propria-infiltrating immune cell subsets, and to explore their association with ustekinumab-induced clinical response and remission in patients.

## 2. Materials and Methods

### 2.1. Study Design and Setting

This was a prospective, observational, single-center, open-label study conducted at the Inflammatory Bowel Disease (IBD) Unit, Policlinico Universitario di Monserrato (Cagliari, Italy). The study was designed to investigate whether baseline mucosal expression of IL-12/IL-23 cytokine subunits and their receptors in lamina propria mononuclear cells (LPMCs) was associated with clinical outcomes after ustekinumab (UST) therapy.

### 2.2. Patient Population

Consecutive adult patients with a confirmed diagnosis of CD were enrolled between April 2019 and May 2024. Patients were required to have moderate-to-severe active disease, with clinical indication to start ustekinumab therapy according to national/international guidelines [8,9], and be willing to undergo ileo-colonoscopy with biopsy sampling.

Key exclusion criteria included inadequate tissue sampling for subsequent immunological analyses (samples were excluded when LPMC isolation and/or RNA extraction quality was insufficient), concomitant use of steroids (including both systemic and low-bioavailability/topical formulations), patients who were not eligible for endoscopic evaluation, and those who were unable to understand and provide informed consent.

### 2.3. Treatment and Follow-Up

At baseline, clinical disease activity was assessed according to the Harvey–Bradshaw Index (HBI) and inflammatory biomarkers including C-reactive protein (CRP) and fecal calprotectin (FC) were collected. All patients underwent ileo-colonoscopy and biopsy sampling. Endoscopic activity was reported using the Simple Endoscopic Score for Crohn’s Disease (SES-CD) and biopsy specimens were collected from the most inflamed areas. Patients received ustekinumab according to standard clinical practice. Clinical outcomes were assessed at baseline and at the end of the induction (week 16). Patients were categorized as clinical responders or non-responders based on clinical improvement at week 16 (defined as a reduction of at least 3 points in the HBI compared with baseline). Responders were further categorized as remitters (Rs) vs. non-remitters (NRs) with clinical remission defined as an HBI score ≤ 4 at week 16.

### 2.4. Biopsy Collection and LPMC Isolation

During baseline ileo-colonoscopy, multiple biopsies were obtained from the most inflamed mucosal areas. Biopsy specimens were used for isolation of lamina propria mononuclear cells (LPMCs) for quantitative real-time PCR and flow cytometry immunophenotyping. LPMCs were isolated from mucosal biopsies using a sequential chelation and enzymatic digestion protocol, as previously described by Morral et al. [10]. Briefly, biopsies were first incubated in an EDTA-containing buffer to induce calcium chelation and detach the epithelial layer, followed by mechanical agitation. The remaining tissue was subsequently subjected to enzymatic digestion with Liberase TM and DNase I (Roche, Basel, Switzerland) to release lamina propria cells. The resulting cell suspension was filtered, washed, and resuspended in complete medium prior to downstream applications. Cell viability and yield were assessed before immunophenotyping.

### 2.5. Quantitative Real-Time PCR

Quantitative real-time PCR (qPCR) was performed to assess baseline mucosal expression of IL-12 and IL-23 cytokine subunits and their receptors. Total RNA was extracted from biopsy-derived LPMCs using an Invitrogen RNA extraction kit, according to the manufacturer’s instructions. RNA concentration and purity were assessed spectrophotometrically. Complementary DNA (cDNA) was synthesized from total RNA using an Invitrogen reverse transcription kit following the manufacturer’s protocol.

Gene expression of IL12(p40) and IL12(p35) was quantified using pre-designed TaqMan Gene Expression Assays (Thermo Fisher Scientific, Waltham, MA, USA; IL12B Hs01011518_m1 and IL12A Hs00168405_m1). All other targets were quantified using SYBR Green chemistry with gene-specific primers and the Luna Universal SYBR Green qPCR Master Mix (New England Biolabs, Ipswich, MA, USA). Real-time PCR reactions were performed on a OneStep Real-Time PCR System using standard cycling conditions according to the manufacturers’ recommendations. Each sample was run in duplicate, and no-template controls were included in every run.

The following primer sequences were used for SYBR Green-based amplification: β-actin was used as the housekeeping gene (forward 5′-AAGATGACCCAGATCATGTTTGAGACC-3′ and reverse 5′-AGCCAGGTCCAGACGCAGGAT-3′); IL23-p19 forward 5′-GGGACACATGGATCTAAGAG-3′ and reverse 5′-GCAAGCAGAACTGACTGTTG-3′; IL12RB1 forward 5′-ACTTCCTGCGGTGTTGCCTT-3′ and reverse 5′-TTCCACCCAGAGTGTGACAGT-3′; IL12RB2 forward 5′-AATTGGATGGCGTTTGTGGC-3′ and reverse 5′-AAGGGCAGCTGTGTCTTCTC-3′; and IL23R forward 5′-GCAGCCTTGGAGTTCACTGT-3′ and reverse 5′-GCCCTGTAGAGATGGAAGCA-3′.

Relative gene expression was calculated using the comparative Ct (2^−ΔΔCt^) method after normalization to β-actin and expressed as relative expression units.

### 2.6. Flow Cytometry Immunophenotyping

Multiparametric flow cytometry was performed on freshly isolated LPMCs obtained from baseline mucosal biopsies. Cells were stained with a viability dye and fluorochrome-conjugated monoclonal antibodies specific for T helper and innate lymphoid cell markers, as well as for IL-12 and IL-23 receptor subunits (reported in Supplementary Table S1). Stained cells were acquired on a MoFlo Astrios EQ cell sorter (Beckman Coulter, Brea, CA, USA) configured for high-parameter analysis, and data were collected using the instrument’s acquisition software. All flow cytometry files were subsequently analyzed using FlowJo software (version 10, BD Biosciences, Franklin Lakes, NJ, USA), applying a unified gating strategy for the identification of T helper and innate lymphoid cell subsets as described below. Lamina propria mononuclear cells (LPMCs) were analyzed by multiparametric flow cytometry. The complete gating strategy is shown in Supplementary Figure S1.

T helper cells were identified through a sequential gating strategy. Briefly, lymphocytes were first selected based on forward and side scatter properties, followed by exclusion of doublets and dead cells. Viable CD45^+^ leukocytes were then gated and CD3^+^CD4^+^ cells were identified as total T helper cells. Within the CD3^+^CD4^+^ population, Th1, Th17 and Th1/Th17 subsets were defined according to CXCR3 and CCR6 expression, as previously described [11,12]. Within each subset, surface expression of IL-23R and IL-12Rβ2 was assessed and reported as the percentage of receptor-positive cells.

For innate lymphoid cell (ILC) immunophenotyping, LPMCs were stained with antibodies against CD45, a lineage cocktail, CD127, IL-23R and IL-12Rβ2. After exclusion of doublets, viable CD45^+^ cells were selected and ILCs were identified as lineage-negative CD127^+^ lymphocytes, as previously described [13]. Within the ILC gate, surface expression of IL-23R and IL-12Rβ2 was quantified and expressed as the percentage of positive cells. All samples were acquired using identical instrument settings and analyzed using the same gating strategy. Fluorescence minus one (FMO) controls were used to define positive gates.

### 2.7. Statistical Analysis

Continuous variables were summarized as means (range), and categorical variables as counts and percentages. Baseline immunological variables (qPCR targets and flow cytometry frequencies/receptor expression) were compared between outcome groups (remitters vs. non-remitters). Two-group comparisons were performed using non-parametric tests (Mann–Whitney U) when distributional assumptions were not met, and categorical variables using Fisher’s exact test. A two-sided *p*-value < 0.05 was considered statistically significant.

Given the exploratory nature of the study and the evaluation of multiple qPCR and flow cytometry endpoints, a post hoc multiple comparison correction was performed using the Benjamini–Hochberg false discovery rate (FDR) procedure. FDR-adjusted *p*-values were calculated as a sensitivity analysis to assess the robustness of the observed associations and were interpreted in the context of the hypothesis-generating design of the study.

### 2.8. Ethics

The study was conducted in accordance with the Declaration of Helsinki and approved by the local Ethics Committee Fondazione PTV, n 191/18, issued on 3 December 2018. All participants provided written informed consent prior to inclusion and sampling.

## 3. Results

### 3.1. Patient Population and Clinical Outcomes

Between April 2019 and May 2024, a total of 16 patients with moderate-to-severe CD were enrolled in the study. One patient was excluded due to inadequate biopsy sampling, leaving 15 patients for the final analysis. Baseline demographic and clinical characteristics of the study population are summarized in Table 1. At week 16, 14/15 (93.3%) patients achieved clinical response and 9/15 (60.0%) achieved clinical remission. Among the 14 responders, 9 (64.3%) fulfilled the criteria for clinical remission, whereas 5 (35.7%) showed clinical improvement without reaching remission.

### 3.2. Baseline Mucosal Expression of IL-12, IL-23 and Their Receptors

**Figure 1:**
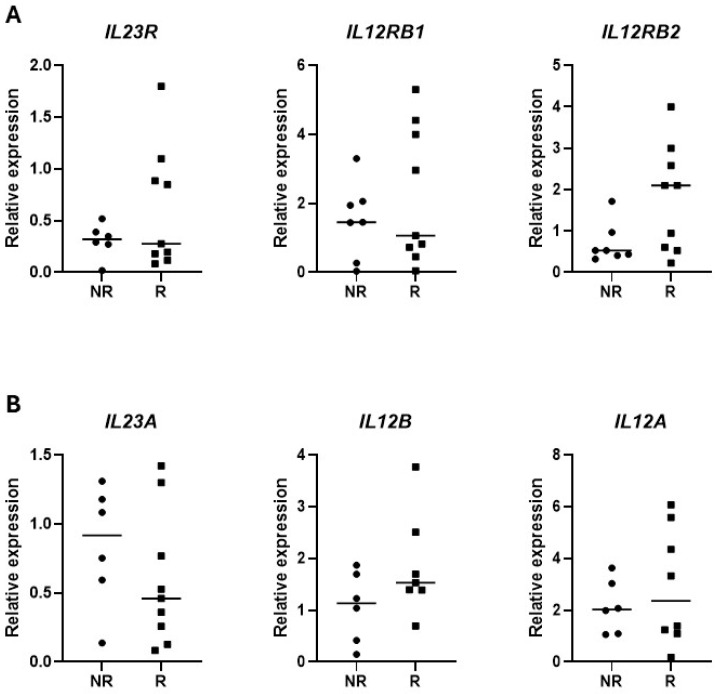
Baseline mucosal gene expression of IL-12/IL-23 pathway components in lamina propria mononuclear cells from patients with Crohn’s disease who achieved or did not achieve clinical remission following ustekinumab therapy. (**A**) Relative expression of *il23r, il12rb1* and *il12rb2* in lamina propria mononuclear cells from remitters (Rs) and non-remitters (NRs). (**B**) Relative expression of *il23a* (p19), *il12a* (p35) and *il12b* (p40) cytokine subunits in remitters and non-remitters. Each dot represents an individual patient. Horizontal bars indicate median values. Gene expression levels were normalized to β-actin and expressed as relative expression.

Baseline mRNA mucosal expression of IL-12 and IL-23 and their receptors was assessed by RT-qPCR on LPMCs isolated from intestinal biopsies. Since clinical response at week 16 was observed in all but one of the 15 patients analyzed, all the association analysis was performed considering week 16 clinical remission. As shown in Figure 1A, no statistically significant differences were observed between remitters (Rs) and non-remitters (NRs) in the expression of the receptor subunits *il12rb1*, *il12rb2* and *il23r*. However, a trend toward higher expression of these receptor subunits was observed in remitters, with *il23r* (R 0.61 ± 0.59 vs. NR 0.31 ± 0.17), *il12rb1* (R 2.20 ± 1.98 vs. NR 1.50 ± 1.11) and *il12rb2* (R 1.79 ± 1.29 vs. NR 0.70 ± 0.49), mainly driven by clusters of outlier patients.

Similarly, no statistical difference was observed in the expression of IL12 and IL23 cytokine subunits (Figure 1B). However, a trend of lower expression of *il23-p19* was observed in remitters as compared to the patient who did not reach remission (R 0.59 ± 0.48 vs. NR 0.84 ± 0.44).

Owing to the limited sample size, no clear transcriptional pattern emerged that could reliably discriminate patients according to clinical outcome following ustekinumab therapy. However, these data suggest that, at least in part, response to UST might be associated with the upregulation of IL23R signaling in specific LPMC subsets.

### 3.3. Distinct Expression of IL-23R, but Not IL-12R, in LPMC Subsets Is Associated with Ustekinumab-Induced Remission

**Figure 2:**
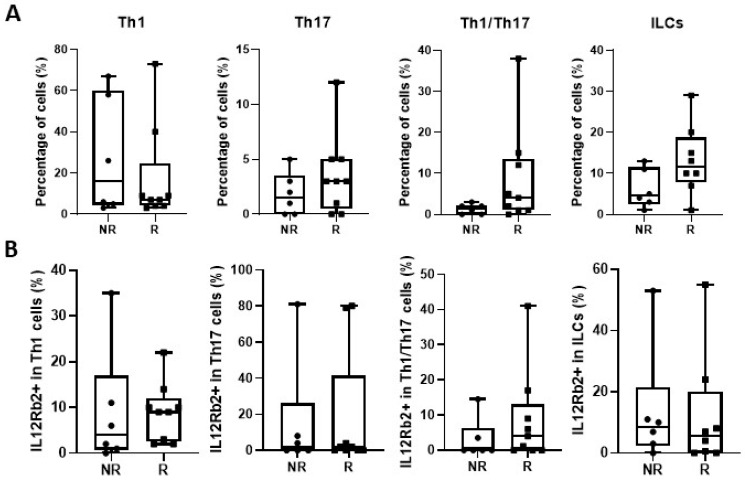

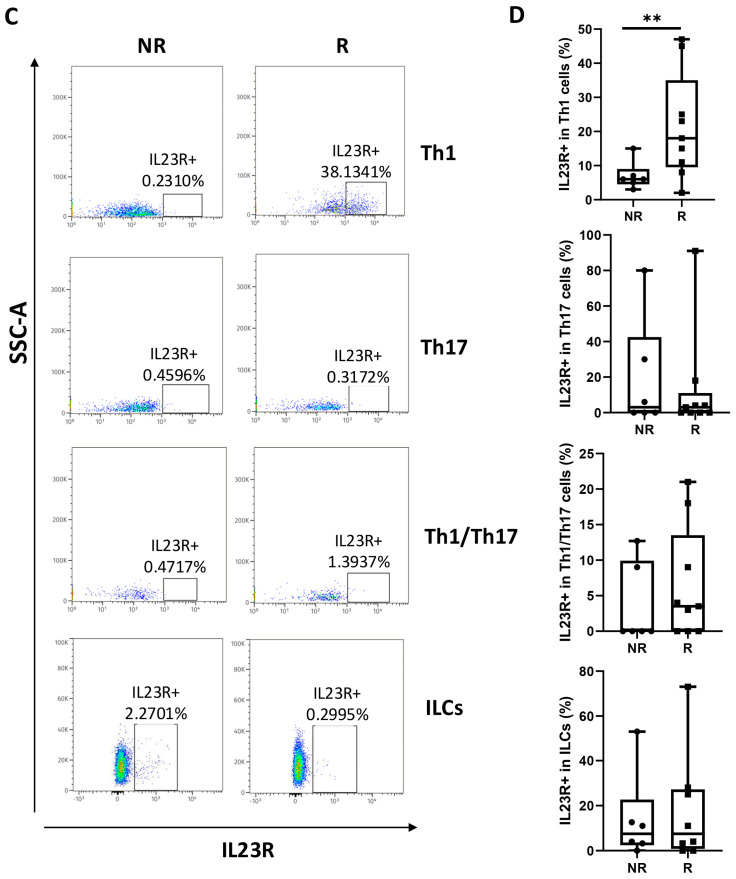
Baseline mucosal immunophenotype and IL-12/IL-23 receptor expression on lamina propria lymphocyte subsets from patients with Crohn’s disease who achieved or did not achieve clinical remission following ustekinumab therapy. (**A**) Frequency of Th1, Th17, Th1/Th17 and ILC1 subsets among lamina propria lymphocytes at baseline in remitters (Rs) and non-remitters (NRs). (**B**) Percentage of IL12Rβ2-positive cells within each indicated subset. (**C**) Representative dot plots of lamina propria mononuclear cells from non-remitters (NRs) and remitters (Rs) showing IL-23R expression on CXCR3^+^CCR6^−^ (Th1), CXCR3^−^CCR6^+^ (Th17), CXCR3^+^CCR6^+^ (Th1/Th17) CD4^+^ T cell and Lin-CD127^+^ (ILC) subsets. (**D**) Percentage of IL23R-positive cells within each indicated subset. IL23R expression on Th1 cells was significantly higher in remitters compared with non-remitters (** *p* = 0.009). Data are shown as box-and-whisker plots with median and interquartile range.

Since IL23 signaling elements can be differentially expressed in lamina propria cell subsets, we first performed immunophenotyping of lamina propria mononuclear cells isolated from baseline biopsies by multiparametric flow cytometry. As shown in Figure 2A, baseline frequencies of Th1, Th17, Th1/Th17, and ILC1 subsets, known to respond to IL23 signaling, were comparable between Rs and NRs, with no statistically significant differences between the two groups.

At the subset level, expression of IL-12Rβ2 on Th1, Th17, and Th1/Th17 cells and ILC1 did not differ significantly between Rs and NRs (Figure 2B). Similarly, the expression of IL-23R on Th17 and ILC1 was comparable between the two groups (Figure 2C,D). In contrast, a marked difference was observed in IL-23R expression on mucosal Th1 cells, which was significantly higher in Rs compared with NRs (mean R 20.6% vs. NR 6.8%, *p* = 0.009; Figure 2C,D). Higher expression of IL23R was also observed in the Th1/Th17 isolated from R patients (mean R 6.5% vs. NR 3.6%, *p* = 0.387). However, the variability observed in this small group of patients did not reach statistical significance.

These data suggest that enhanced IL-23R expression in lamina propria Th1 cells may be associated with a more favorable response to the anti-IL-12/IL-23 p40 monoclonal antibody ustekinumab. However, when the exploratory flow cytometry comparisons were adjusted for multiple testing using the Benjamini–Hochberg false discovery rate procedure, this association did not retain formal statistical significance. Therefore, these findings should be considered exploratory and hypothesis-generating, warranting validation in larger independent cohorts before any definitive conclusions can be drawn.

## 4. Discussion

In this study, we characterized the baseline mucosal expression of IL-12 and IL-23 and that of their receptors in lamina propria cell subsets isolated from patients with CD treated with ustekinumab, identifying a distinct signature associated with clinical remission.

Consistent with the central role of the IL-12/IL-23 axis in intestinal inflammation, both IL-12 and IL-23 have been implicated in the pathogenesis of CD through their influence on adaptive immune responses, particularly Th1 and Th17 cell pathways. IL-23 is a key proinflammatory cytokine that promotes the survival and expansion of pathogenic T cell subsets and modulates innate and adaptive immunity in the gut mucosa [14].

Ustekinumab, a monoclonal antibody targeting the shared p40 subunit of IL-12 and IL-23, has demonstrated efficacy in moderate-to-severe CD, including in patients with prior anti-TNF exposure, but predictors of response remain poorly defined [7]. Traditional analyses of bulk mucosal expression of IL-12 and IL-23 pathway components did not reveal significant differences between remitters and non-remitters in our cohort, potentially reflecting both the complexity of cytokine networks in the gut and the limited sample size [6]. Consistent with these findings, transcriptional analysis of bulk mucosal IL-12/IL-23 receptors in our cohort showed no statistically significant differences between remitters and non-remitters, suggesting that transcriptional profiling alone may be insufficient to capture the functional diversity of immune cell subsets relevant to therapeutic response. This apparent discrepancy may reflect the heterogeneous cellular composition of lamina propria mononuclear cells. Indeed, transcriptional changes confined to relatively small but biologically relevant immune subsets, such as IL-23R-expressing Th1 cells, may be diluted in bulk RNA analyses while remaining detectable by subset-specific flow cytometry.

In contrast, our immunophenotyping revealed a significant enrichment of IL-23R-expressing Th1 cells at baseline in patients achieving clinical remission with ustekinumab. This observation aligns with emerging evidence that IL-23 can influence not only classical Th17 pathways but also pathogenic T cell populations with mixed effector profiles. IL-23 signaling has been shown to support the survival and effector functions of memory T cells and to drive a proinflammatory phenotype in cells that co-express IFN-γ and IL-23R [15]. Experimental models have demonstrated that IL-23R expression on Th1-like cells contributes to their colitogenic potential and that IL-23R signaling can shape the gene expression profile of pathogenic T cells in ways distinct from classical Th17 cells [3]. Importantly, surface expression of IL-23R should not be equated with functional activation of the IL-23 signaling pathway. Since downstream signaling events, such as STAT3 phosphorylation, cytokine production, or ex vivo responses to IL-23 stimulation, were not assessed in the present study, the functional responsiveness of IL-23R^+^ Th1 cells remains to be established. These results also resonate with prior mechanistic hypotheses regarding anti-TNF therapy failure. Schmitt et al. reported that non-responders to TNF-α antagonists exhibit expansion of apoptosis-resistant TNFR2^+^IL23R^+^ mucosal T cells and heightened mucosal IL-23p19 expression during anti-TNF treatment, suggesting that IL-23-driven pathways may sustain inflammation when TNF signaling is blocked [3]. Their findings provide a biological rationale for the observation that some patients fail to respond to TNF blockade due to activation of alternative proinflammatory circuits dominated by IL-23. Our data extend these insights by suggesting that the baseline abundance of IL-23R-positive Th1 cells may identify a subset of patients more likely to derive benefit from ustekinumab, which directly interferes with IL-23 signaling. These findings suggest that IL-23R expression on mucosal Th1 cells may represent a promising candidate biomarker associated with treatment outcome; however, prospective validation and mechanistic studies are required before any predictive role can be established.

Our study has several limitations, including the relatively small sample size and the observational single-center design, which may limit the generalizability of the findings. In addition, baseline differences in disease behavior, inflammatory burden, and prior biologic exposure may have influenced treatment outcomes. Given the limited sample size and the small number of remission events, the study was not adequately powered to adjust for these potential confounders. This study was performed during the COVID-19 pandemic and the limited access of patients to our clinic, the limitation in performing endoscopy if not strictly necessary, and restricted access to our laboratories to perform the analysis of human biopsy samples heavily affected our capacity to enroll an adequate number of patients in the study.

Moreover, the exploratory evaluation of multiple immunological endpoints increases the possibility of type I error. To address this issue, we performed a post hoc Benjamini–Hochberg false discovery rate correction. Although the association between baseline IL-23R expression on Th1 cells and clinical remission did not retain formal statistical significance after adjustment, the observed biological trend remains of interest and should be considered hypothesis-generating. The limited sample size of this pilot study may also have reduced the statistical power after correction for multiple testing. Additional limitations include the absence of healthy controls and the use of clinical remission based on the HBI as the primary outcome. Future studies should incorporate objective endoscopic and histological endpoints to better validate the predictive value of the identified immunological signatures. Therefore, these findings require validation in larger prospective cohorts before any clinical application.

## 5. Conclusions

In conclusion, increased baseline expression of IL-23R on mucosal Th1 cells emerged as an exploratory immunological feature associated with clinical remission following ustekinumab therapy in Crohn’s disease. This finding should be considered hypothesis-generating and warrants validation in larger prospective studies before IL-23R can be regarded as a predictive biomarker.

## Figures and Tables

**Table 1:** Baseline demographic and clinical characteristics of patients with Crohn’s disease included in the study.

Baseline Characteristics of Patients with Crohn’s Disease	N = 15
Male n (%)	10 (66.7%)
Mean age (range)	55.8 (31–79)
Mean duration of disease yrs (range)	19.6 (10–32)
Previous anti-TNF exposure n (%)	
IFX	6 (40%)
ADA	11 (73.3%)
Disease characteristics n (%)	
Disease location	
L1	8 (53.3%)
L2	3 (20%)
L3	3 (20%)
P	4 (26.6%)
Disease behavior	
B1	5 (33.3%)
B2	6 (40%)
B3	4 (26.7%)
CRP (mg/L) mean (range)	12.2 (0.2–90)
FC (µg/g) mean (range)	930 (55–2300)
HBI mean (range)	10 (17–4)
SES-CD mean (range)	10 (5–14)

Abbreviations: IFX, infliximab; ADA, adalimumab; CRP, C-reactive protein; FC, fecal calprotectin; HBI, Harvey–Bradshaw Index; SES-CD, Simple Endoscopic Score for Crohn’s Disease; L1, ileal disease; L2, colonic disease; L3, ileocolonic disease; P, perianal disease; B1, non-stricturing, non-penetrating disease; B2, stricturing disease; B3, penetrating disease.

## Data Availability

All data generated or analyzed during this study are included in this published article and its Supplementary Materials.

## Supplementary Materials

The following supporting information can be downloaded at: https://www.mdpi.com/article/10.3390/jcm15145434/s1, Figure S1: Flow cytometry gating strategy for identification of T helper cell subsets and innate lymphoid cells in lamina propria mononuclear cells. Table S1: Panel used for immunophenotyping of T helper cells and innate lymphoid cells.
