# Supplementary material for: Patients with Crohn’s Disease Achieving Ustekinumab-Induced Remission Are Characterized by Increased Baseline IL-23 Receptor Expression on Lamina Propria Th1 Cells
Source: J Clin Med. 2026 Jul 10;15(14):5434. doi: 10.3390/jcm15145434 (PMC13412810; doi:10.3390/jcm15145434)
Supplement: Supplementary file 1 [file jcm-15-05434-s001.zip › jcm-4353398-supplementary.pdf]

Supplementary Table 1. Panel used for immunophenotyping of T helper cells and innate lymphoid cells

Marker	Clone	Fluorochrome	Brand
Live/Dead	—	V450	Invitrogen
CD45	J33	Krome Orange	Beckman Coulter
CD3	OKT3	PerCP-Cy5.5	eBioscience
CD4	RPA-T4	APC-Cy7	BioLegend
CCR6	R6H1	FITC	eBioscience
CXCR3	1C6	Alexa Fluor 700	BD Biosciences
IL23R	218213	APC	R&D Systems
IL12Rβ2	2B6/12beta2	PE	BD Biosciences
Lineage cocktail	—	FITC	BD Biosciences
CD127	RDR5	Alexa Fluor 700	eBioscience
CD161	HP-3G10	PerCP-Cy5.5	eBioscience

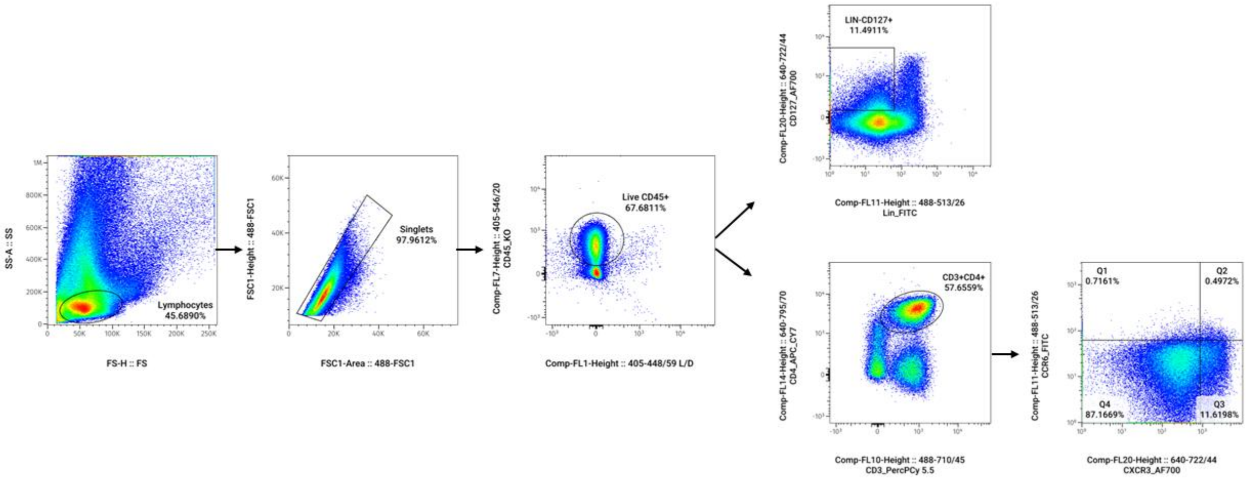

Supplementary Figure 1. Flow cytometry gating strategy for identification of T helper cell subsets and innate lymphoid cells in lamina propria mononuclear cells.

Sequential gating showing identification of lymphocytes based on forward and side scatter properties, exclusion of doublets, selection of viable $CD45^+$ leukocytes, and gating on $CD3^+CD4^+$ T helper cells. $CD4^+$ T cells were further subdivided according to CXCR3 and CCR6 expression using quadrant gating: Q1 ($CXCR3^-$CCR6+, Th17), Q2 ($CXCR3^+$CCR6+, Th1/Th17), and Q3 (CXCR3+CCR6-, Th1). In parallel, innate lymphoid cells were identified by gating on lineage-negative $CD45^+$ lymphocytes and selecting Lin^-CD127^+ cells. IL-12Rβ2 and IL-23R surface expression was analyzed within the gated $CD4^+$ T-cell subsets (Q1–Q3) and within the ILC gate. Gates were defined using fluorescence minus one (FMO) controls.
